# Oxidative Stress and DNA Damage in Human Gastric Carcinoma: 8-Oxo-7′8-dihydro-2′-deoxyguanosine (8-oxo-dG) as a Possible Tumor Marker

**DOI:** 10.3390/ijms14023467

**Published:** 2013-02-06

**Authors:** Silvia Borrego, Antonio Vazquez, Francisco Dasí, Concha Cerdá, Antonio Iradi, Carmen Tormos, Julia M. Sánchez, Leticia Bagán, Javier Boix, Cristóbal Zaragoza, Jordi Camps, Guillermo Sáez

**Affiliations:** 1 Clinical Analysis Service, CDB-General University Hospital, Avda. Tres Cruces s/n., Valencia 46014, Spain; E-Mails: silvictbo@gmail.com (S.B.); cerda_con@gva.es (C.C.).; 2 Oxidative Stress Research Group, SEQC, Calle Padilla 323, Barcelona 08025, Spain; 3 Department of General Surgery and Digestive Diseases, General University Hospital, Avda. Tres Cruces s/n, Valencia 46014, Spain; E-Mails: vprado.a@gmail.com (A.V.); zaragoza_cri@gva.es (C.Z.); 4 Valencia Clinical Hospital Research Foundation (INCLIVA), Avda. Blasco Ibáñez 15, Valencia 46010, Spain; E-Mail: Francisco.Dasi@uv.es; 5 Department of Physiology, Faculty of Medicine, University of Valencia, Avda. Blasco Ibañez 15, Valencia 46010, Spain; E-Mail: antonio.iradi@uv.es; 6 Department of Biochemistry and Molecular Biology, Faculty of Medicine, University of Valencia, INCLIVA. Avda. Blasco Ibañez 15, Valencia 46010, Spain; E-Mails: mctormos@uv.es (C.T.); julmst@gmail.com (J.M.S.); leticiabagan@hotmail.com (L.B.); 7 Department of Pathology, Faculty of Medicine, University of Valencia, Avda. Blasco Ibañez 15, Valencia 46010, Spain; E-Mail: javier.boix@uv.es; 8 Bimedical Research Center, Sant Joan University Hospital, Health Institute of Research Pere Virgili, Rovira i Virigili University, Reus 43201, Spain; E-Mail: jcamps@grupsagessa.com

**Keywords:** 8-oxo-dG, DNA repair enzymes, gastric cancer, oxidative stress, tumor marker

## Abstract

We characterized the oxidative stress (OS) status by the levels of reduced/oxidized glutathione (GSH/GSSG), malondialdehyde (MDA) and the mutagenic base 8-oxo-7′8-dihydro-2′-deoxyguanosine (8-oxo-dG) in human gastric carcinoma (HGC) samples and compared the results with normal tissue from the same patients. We also analyzed 8-oxo-dG in peripheral mononuclear cells (PMNC) and urine from healthy control subjects and in affected patients in the basal state and one, three, six, nine and twelve months after tumor resection. The levels of DNA repair enzyme mRNA expression (hOGG1, RAD51, MUYTH and MTH1) were determined in tumor specimens and compared with normal mucosa. Tumor specimens exhibited increased levels of MDA and 8-oxo-dG compared with normal gastric tissue. GSH levels were also increased, while GSSG levels remained stable. DNA repair enzyme mRNA expression was induced in the tumor tissues. Levels of 8-oxo-dG were significantly elevated in both urine and PMNC of gastric cancer patients compared with healthy controls. After gastrectomy, the levels of the damaged base in urine and PMNC decreased progressively to values close to those found in the healthy population. The high levels of 8-oxo-dG in urine may be related to the increased induction of DNA repair activity in tumor tissue, and the changes observed after tumor resection support its potential use as a tumor marker.

## 1. Introduction

Living cells generate reactive oxygen species (ROS) under physiological conditions as a result of their aerobic metabolism. Monovalent reduction of oxygen is responsible for the production of intermediates, such as hydrogen peroxide (H_2_O_2_) and superoxide radicals (O_2_^−^), which give rise to hydroxyl radicals (*OH) through Haber–Weiss or Fenton-type reactions. Due to their paramagnetic configuration, O_2_^−^ and *OH are highly reactive and have strong cytotoxic properties. However, a number of regulatory functions are also attributed to ROS molecules [1].

The damaging effects induced by ROS and by other free radicals include a number of oxidative modifications and/or alterations of critical molecules in a complex reactive scheme, which is defined as oxidative stress (OS) [2]. Molecular targets of ROS include unsaturated phospholipids, proteins, carbohydrates and nucleic acids (DNA or RNA). Consequently, the structure and viability of cell functions become compromised. Therefore, it is generally accepted that OS is involved in the physiopathology of degenerative diseases, including the ageing process and cancer. Oxidative stress by-products are increased in the blood and tissues of affected patients, and considerable efforts have been made to validate their roles as clinical markers [3–5]. However, the predictive value of these markers is still an unresolved problem, and further investigations are required [6,7].

Oxidative DNA damage is a result of attack by ROS, and a number of damage DNA bases can be measured by various methods in total DNA and in urine after its repair by specific enzyme mechanisms [1,3]. Urinary 8-oxo-dG reflects the equilibrium between its production and repair in both DNA and the nucleotide pool. Many DNA repair mechanisms take place in cells to correct all the different forms of damage that may occur [8,9]. Some DNA repair enzymes are specific to substrates, such as hOGG1 for the removal of 8-oxo-dG [10], MTH1 for the hydrolysis of 8-oxo-GTP in the nucleotide pool and the proofreading enzyme [11] MTH1 for the excision of adenine opposite 8-oxo-dG [12]. Others are involved in the homologous recombination of double-strand DNA breaks, such as the RAD51 protein [13]. Repair of oxidized guanine in both DNA and the nucleotide pool is an essential feature for the maintenance of cell homeostasis, as 8-oxo-dG is a mutagenic base that can form a mismatch pair with A leading to GC→TA transversion [14,15]. Therefore, increased levels of 8-oxo-dG in DNA may contribute to gene instability, affecting the normal function of oncogenes and tumor suppressor genes in the pathogenesis of tumor initiation and promotion [16–19]. Additionally, many human tumors exhibit significantly elevated levels of the mutagenic base 8-oxo-dG in comparison with unaffected cells [19–22]. Elevated levels of urinary 8-oxo-dG have also been reported in cancer patients [23,24], suggesting stable or even enhanced DNA damage repair mechanisms. Such increased ROS production may induce the transcription or post-translational modification of base excision and other DNA repair enzymes [25,26].

Human gastric cancer is a common disease and one of the leading causes of cancer mortality worldwide. Gastric adenocarcinoma accounts for more than 95% of gastric tumors [27]. Sporadic gastric tumors are known to be related to a variety of etiological factors, such as diet, alcohol and tobacco habits, as well as *Helicobacter pylori*-induced inflammation, all of which may be related to underlying production of ROS and DNA damage [28]. The generation and increase of OS and secondary DNA oxidative damage are also known to be related to the damage and malignant transformation of gastric mucosa [29]. Additionally, increased expression of human DNA repair genes has been reported in digestive tract tumors [30,31].

Clarifying the mechanisms of the genesis, metabolism and physiological properties of OS by-products may be important to identify new biomarkers. The study of endogenous mechanisms of carcinogenesis by measuring oxidative markers has advanced greatly over the past two decades, paralleling similar achievements in exogenous carcinogenesis through the analysis of DNA adducts [32]. Although the role of OS and its implications in genetic alterations inducing human tumorigenesis has been extensively investigated, efforts to identify oxidation damage products as clinical markers have been inconclusive. We monitored the yield of damaged bases in urine and peripheral mononuclear cell (PMNC) DNA of gastric cancer patients before and after gastrectomy. We also analyzed induction of the expression of DNA repair enzymes to investigate the origin of the increased levels of urinary 8-oxo-dG in gastric carcinoma patients. High levels of DNA damage products in urine and PMNC of affected patients and their time-dependent decrease were correlated with the normal population values. This is the first experimental study to characterize the OS status in gastric tumors, PMNC and urine of affected patients, and the results suggest 8-oxo-dG is a possible tumor marker.

## 2. Results and Discussion

### 2.1. Oxidative Stress Status in Gastric Tumor Patients

**Figure 1:**
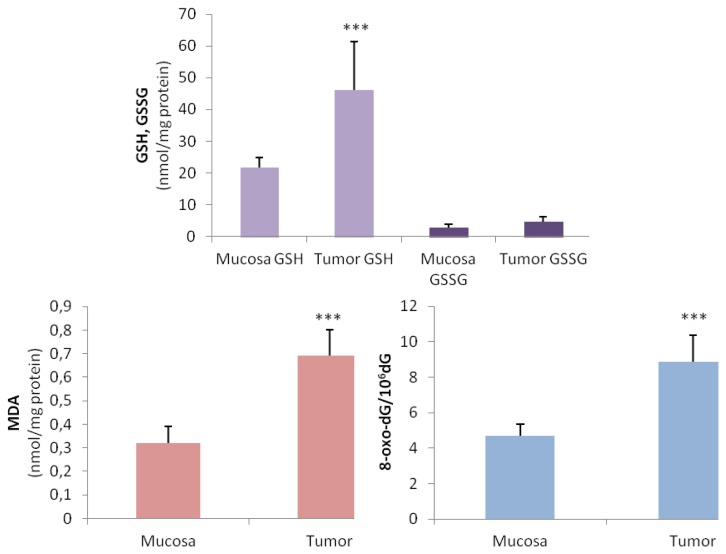
Oxidative stress by-products in gastric carcinoma *vs.* normal mucosa. Tissues were obtained as outlined in the Experimental Section and dealt with appropriately for metabolite assays. A total of 28 paired samples were used. Results are expressed as means ± SD. *** *p* < 0.001 in the comparison between tumor and normal mucosa.

We characterized OS status by measuring the most representative indicators, such as glutathione levels, MDA and 8-oxo-dG, in tumor samples from affected patients and compared them with the corresponding non-affected mucosa. As shown in Figure 1, significant increases in GSH level were observed in tumor tissue compared with normal mucosa from the same patients. The levels of GSSG in the tumor samples were also increased, but the differences were not significant. Malondialdehyde levels were significantly increased in gastric tumors, suggesting an increase in lipid peroxidation status in the affected tissue (0.32 nmol/mg protein in normal mucosa *vs.* 0.67 nmol/mg protein in tumor tissue). A significant amount of 8-oxo-dG was identified in the DNA of gastric carcinoma tissue compared with normal mucosa (4.3 8-oxo-dG/10^6^ dG in healthy tissue *vs.* 7.99 8-oxo-dG/10^6^ dG in tumor tissue).

Furthermore, 8-oxo-dG was significantly increased in the urine and PMNC of gastric carcinoma patients compared with the established values for healthy subjects. The 8-oxo-dG levels in the urine of affected patients were increased by about tenfold. Significant increases were also observed in intracellular MDA levels, while GSH levels were decreased in these cells in comparison with the control group. The concentration of GSSG was not significantly affected, resulting in a significant increase in the GSSG/GSH ratio (Table 1).

### 2.2. DNA Repair Enzyme mRNA Expression

**Figure 2:**
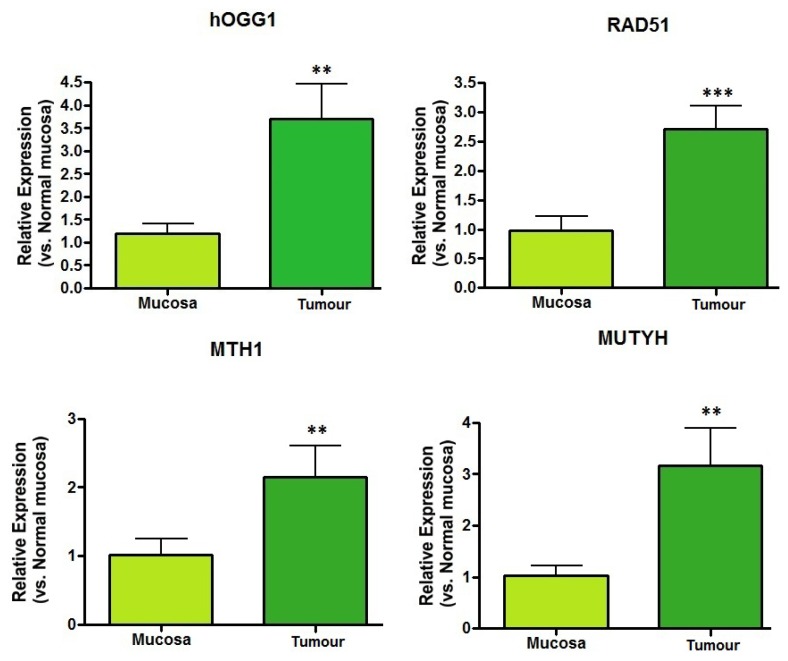
mRNA expression of DNA repair enzymes in tumor tissue and normal mucosa. The figure presents relative mRNA expression levels for the indicated enzymes and their significance. Glyceraldehyde-3-phosphate dehydrogenase (GAPDH) expression was analyzed to normalize gene expression for sample-to-sample differences in RNA input, RNA quality and reverse transcriptase efficiency. Each sample was analyzed in triplicate. Results are expressed as means ± SD of ten different tumors and mucosa from the same patient. ** *p <* 0.05; *** *p <* 0.001.

The DNA repair enzymes exhibited increased mRNA expression levels in the tumor specimens in comparison with the basal expression levels of normal tissue samples (Figure 2). The increases in relative mRNA expression levels in the gastric tumors *vs.* normal mucosa were highly significant for hOGG1 (*p <* 0.005), RAD51 (*p <* 0.001), MTH1 (*p <* 0.04) and MUTYH (*p <* 0.01), indicating enhanced DNA repair activity in these tumor cells.

### 2.3. Gastrectomy Time Course Effect on Urinary and PMNC 8-oxo-dG Levels

**Figure 3:**
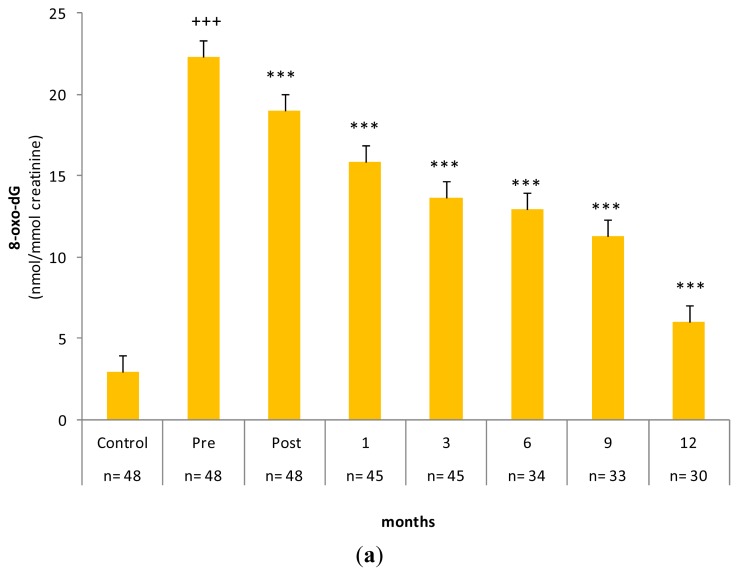

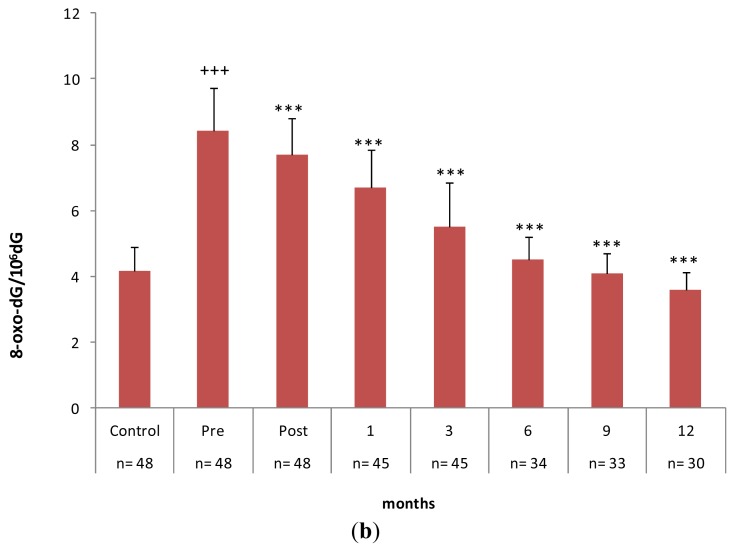
Time course effect of tumor resection on the levels of 8-oxo-dG in (**a**) urine and (**b**) peripheral mononuclear cells of gastric carcinoma patients. Control: healthy subjects; Pre: samples 24 h before surgical intervention; Post: 24 h and following months (1–12) after surgery. Results are expressed as means ± SD with the number of samples (*n*) indicated in the figure. ^+++^*p <* 0.001 for the comparison of control subjects with cancer patients at the basal state; *** *p <* 0.001 for the comparison of post-surgical time periods with basal state in the patient group.

Figure 3a clearly shows the increase in the yield of urinary 8-oxo-dG in the gastric carcinoma group, with a marked difference between patients in the basal state (before surgery) and healthy subjects. After gastrectomy, these values progressively declined and became significant within hours after tumor resection. Over the following months, urinary 8-oxo-dG levels further decreased and reached a significantly reduced value compared to the basal state. A similar pattern of differences and changes in 8-oxo-dG levels was observed in PMNC. The level of the damaged base in the PMNC of tumor patients was twice that in the control group (Figure 3b and Table 1). After tumor resection, the concentration of 8-oxo-dG in the DNA of PMNC decreased in a time-dependent manner, as observed in the urine of patients. However, in the case of PMNC, 8-oxo-dG levels decreased to equal the levels in the healthy group between six and nine months post-gastrectomy (control group 4.16 ± 0.73 8-oxo-dG/10^6^ dG *vs.* patient group at six months 4.51 ± 0.68 8-oxo-dG/10^6^ dG and at nine months 4.09 ± 0.62 8-oxo-dG/10^6^ dG). A further decrease was observed at 12 months.

In patients undergoing surgical intervention, but without feasible tumor resection due to expansion, the levels of 8-oxo-dG did not change throughout the survival time, which was no longer than three months in the majority of cases. Table 2 lists a representative sample of these results.

### 2.4. Discussion

Representative markers of OS were analyzed to characterize the oxidation status in tumor samples. Malondialdehyde and 8-oxo-dG levels were significantly increased in gastric tumors in comparison with normal mucosa. Reduced glutathione levels were also increased in cancer tissues, consistent with previous research demonstrating that tripeptide levels were significantly elevated in tumor specimens [16–18] and in the blood cells of cancer patients [33,34]. Increased GSH concentration is a common characteristic of tumor cells; this may be related to the increase in GSH synthetase levels found in some tumor cell lines [35] and is responsible for the reduced sensitivity of cancer cells to chemotherapy and radiotherapy [36]. Increased GSH levels may reflect an adaptation against H_2_O_2_ and other peroxides formed in tumor cells. However, in our previous research [19], we found that the increases in glutathione peroxidase and its co-substrate GSH are not sufficient to prevent oxidative stress in cancer cells. Increased GSH levels in gastric tumor cells were not, however, observed in the circulating PMNC of cancer patients, where the opposite effect was observed: a significant reduction in thiol concentration in comparison with the control group (Table 1).

Additionally, the significant increases in MDA and 8-oxo-dG observed in gastric tumors reflect the high OS status of these tissues. Hydroxyl radicals have a strong affinity toward the guanine bases of DNA. Tumor cells are known to exhibit peculiar metabolic characteristics in terms of oxygen consumption and oxidative metabolism. The increased susceptibility of tumor tissues to oxidative stress compared to the surrounding normal cells is supported by the increase in lipid peroxidation and DNA damage and by the decrease in antioxidant enzyme activity [19–22,24,37]. Impairment of antioxidant enzyme activity may explain the accumulation of O_2_^−^ and H_2_O_2_ in tumor cells [38]. This effect may be intensified by the inherent increase of H_2_O_2_ production by transformed cells [39]. An increase in the availability of H_2_O_2_ in the tumor cell milieu may trigger a Haber–Weiss or Fenton-type reaction, leading to the formation of large amounts of *OH and, consequently, to the oxidative modification of guanine in DNA [19]. *OH and, to a lesser extent, the low-energy singlet molecular oxygen (^1^O_2_), through specific targets (guanine, histidine, tryptophan and tyrosine), may react with DNA and proteins. Addition of the hydroxyl radical to the C-8 position of the guanine ring produces an 8-hydroxy-7,8-dihydroguanil radical that can be either oxidized to 8-oxo-7,8-dihydroguanine (8-oxo-Gua) or reduced to give the ring-opened 2,6,-diamino-4-hydroxy-5-formamidopyrimidine (FapyGua) [40]. Augmented 8-oxo-dG levels in either urine or tumor tissue have been reported in cases of acute lymphoblastic leukemia, colorectal cancer, high-grade cervical dysplasia, renal cell carcinoma, many types of lung tumor, prostate cancer, gastric intestinal metaplasia and gastric adenocarcinoma [41].

**Figure 4:**
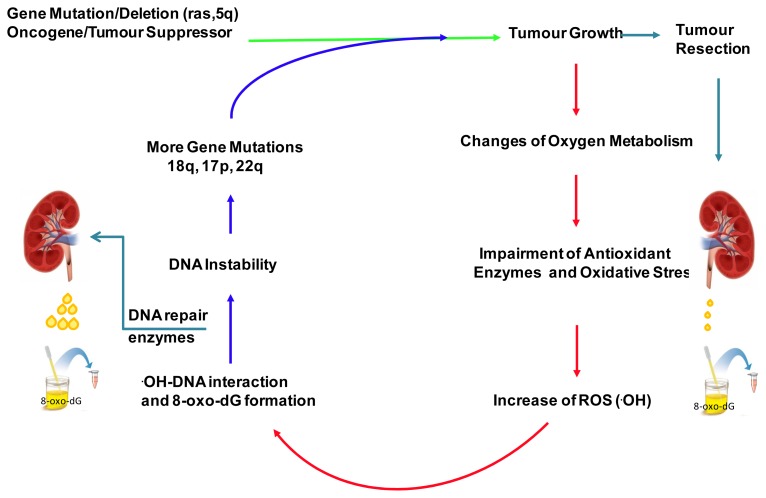
Schematic representation of the roles played by oxidative stress and oxygen free radicals in the induction of genetic instability during tumor progression. Increased oxidative stress and the production of hydroxyl radicals in tumor tissue interact and oxidize DNA, inducing the mutagenic base 8-oxo-dG, leading to increased gene instability in the tumor tissue. Different genetic alterations affecting oncogenes and tumor suppression genes take place during tumor growth and progression, contributing to the pathogenicity of the disease [19]. Induced expression of DNA repair enzymes results in the release of high levels of the damaged base in urine. The 8-oxo-dG levels in the urine of affected patients are reduced after tumor resection.

Oxidative DNA damage has been implicated in DNA instability and the accumulation of genetic mutations affecting oncogenes and tumor suppressor genes [16,17]. A mechanism to explain the ability of ROS to potentiate genomic alterations and gene mutations in tumor cells has been reported previously [19] and is shown in Figure 4. Increased levels of oxidized guanosine, together with elevated hOGG1 activity, have also been reported in colorectal carcinoma tissue [42].

The PMNCs of gastric cancer patients are known to have greater OS as indicated by their by-products, one of which is the damaged base 8-oxo-dG (Table 1). Tumor tissues have a high inflammatory state in which the secretion of a number of diverse chemoattractants induces the recruitment and activation of circulating monocytes. These cells are responsible for the cancer-related inflammation that appears in tumor sites [43]. This mechanism is associated with the production of various oxidants and may substantially stimulate the oxidation of endogenous DNA. A number of observations support the hypothesis that OS, chronic inflammation and cancer are closely related [44]. Recent studies have revealed that a high OS status can be detected in the presence of a tumor or systemic malignancy [45]. Experimental evidence has also indicated that inflammation and/or OS-induced modifications appear not only in the microenvironment surrounding the tumor, but also in distant organs. Redon *et al.* recently suggested that the induction of complex DNA damage by tumors growing in mice is not limited to close proximity, but may involve other proliferative organs through the activation of specific cytokines [46]. Therefore, the contribution of PMNC to the 8-oxo-dG excreted in urine should not be discounted.

It is necessary to clarify the characteristics of the oxidized base 8-oxo-dG in gastric tumors. DNA repair mechanisms are known to be involved in the removal of 8-oxo-dG, which is then concentrated in urine. Thus, 8-oxo-dG has characteristics that make it an excellent indicator of OS *in vivo*, but also as a possible clinical marker [47,48]. However, previous experimental studies have been inconclusive in this respect. We found that most representative DNA repair enzymes are expressed in gastric tumor cells, as demonstrated by the induction of the respective mRNAs. These DNA repair proteins act at different levels to maintain the integrity of the genome [8,9,49].

The most important repair enzyme in mammalian cells relevant to the damaged base 8-oxo-dG is hOGG1 glycosylase [48]; hOGG1 was significantly induced together with RAD51, MUTY and MTH1 in our gastric tumor samples (Figure 2). Increased induction of DNA repair enzymes has also been observed in other human tumors [42,50–55]. A number of experimental assays have indicated that the presence of 8-oxo-dG in urine may reflect the efficiency of repair by specific enzymes [56]. In our study group, the significant increases in 8-oxo-dG in the urine of tumor patients was probably a consequence of the positive induction of DNA repair enzymes acting in different compartments. Moreover, the progressive and constant reduction of this metabolite in urine and PMNC DNA was observed in patients undergoing gastrectomy intervention, but not when surgical removal of the tumor was unsuccessful. These results support the theory that tumor tissue is the principal source of 8-oxo-dG.

Repair of oxidatively damaged DNA is a crucial point in the maintenance of genome integrity, and it is therefore important in the prevention of a wide range of pathological processes, including age-related diseases, such as atherosclerosis and cancer. The availability of non-invasive methods for the assay of 8-oxo-dG makes it feasible to investigate the role of DNA repair efficiency and its pathological implications. To clarify the extent to which DNA lesions are involved in diseases, validation of appropriate analysis methods is essential. A number of experimental approaches have been developed to evaluate oxidative damage to DNA, including gas chromatography with mass spectrometry (GC/MS), high-performance liquid chromatography with electrochemical detection (HPLC-EC), HPLC with single or tandem mass spectrometry, ^32^P-postlabeling, immunoassay, alkaline elution and Comet assay and the nicking of DNA at oxidized bases by repair enzymes [40]. One of the most widespread methods used for determining 8-oxo-dG is HPLC-EC, which is known to render reproducible and accurate results [41,57,58].

8-Oxo-dG is the most-studied urinary biomarker of DNA oxidation, principally because of its mutagenic potential and its probable roles in degenerative diseases, including cancer [3,14–16,28]. It is a pivotal marker for measuring the effects of endogenous oxidative damage to DNA and as an initiation and promotion factor of carcinogenesis [48]. Therefore, its validation as a clinical biomarker deserved special attention. In 1997, the European Standards Committee for Oxidative DNA Damage (ESCODD) was established to resolve the artifactual oxidation problems during the isolation and purification of oxidative DNA products [59].

Some years later, a multicenter research study was carried out to validate the assay and value ranges of 8-oxo-dG in urine [60]. In the present study, HPLC-EC detection enabled us to quantify the levels of 8-oxo-dG in the tumor tissues, PMNC and urine of our gastric carcinoma patients. The values of 8-oxo-dG obtained for the control population were in the range of those reported by other researchers considering the differences in the methodologies used [5,61–64]. Nevertheless, the 8-oxo-dG levels obtained in our control group were slightly higher than those reported by the ESCODD researchers [59], which may have been due to the advanced age of our study population.

Controversial results regarding whether urinary 8-oxo-dG represents the OS status of the body have been reported [65,66]. 8-Oxo-dG is removed from DNA by base excision repair (BER) and is expected to be found in urine as the base 8-oxo-7,8-dihidroguanine. Although nucleotide excision repair (NER) may lead to the production of 8-oxo-dG-containing oligonucleotides and these fragments may be further hydrolysed to release 8-oxo-dG, this pathway has not been demonstrated. At present, although the action of NER and subsequent digestion of oligonucleotide products could theoretically account for a portion of urinary 8-oxo-dG, the pyrophosphohydrolase action of MTH1 on 8-oxo-GTP in cellular nucleotide pools is one of the most likely candidates for the generation of 8-oxo-dG as a repair product and its presence in urine [67]. In our tumor samples, we observed significant induction of the DNA repair enzyme MTH1 (Figure 2). In addition to DNA repair mechanisms, other possible sources of 8-oxo-dG may include diet, cell death and turnover and the cellular uptake and reutilization of damaged products. These potential confounding factors are still under discussion; most researchers agree that their contribution is minimal, but further research is still needed [67].

Identification of novel biomarkers for various cancers and their potential for use in early detection are the most important factors determining the survival of affected patients. The use of tumor markers still has limitations, highlighting the need for the identification of new biomarkers and their validation for cancer prevention. Research has demonstrated that the combined use of different tumor markers in gastrointestinal tumors significantly improves diagnostic accuracy compared to their individual use [68]. The potential use of 8-oxo-dG as a tumor marker could contribute to better diagnosis and follow-up of gastric carcinoma patients. Therefore, the findings of this translational research may improve clinical biochemistry tools for the diagnosis of gastric carcinoma.

## 3. Experimental Section

### 3.1. Recruitment of Subjects and Biological Samples

Twenty-eight gastric adenocarcinoma samples and their corresponding normal mucosa were obtained by surgical resection from patients diagnosed and treated in the Department of General and Digestive Surgery at the General University Hospital of Valencia between October 2009 and January 2012. Histopathological examination was carried out by two independent pathologists on hematoxylin and eosin (H&E)-stained preparations. The histological type and the clinical stage of tumor specimens were classified according to the WHO system [69] and the TNM classification system [70], respectively. Patients included in this study did not receive chemotherapy or radiation before surgery. Cancerous tissue and the corresponding normal mucosal tissue were dissected separately immediately after surgical resection. Control normal mucosal tissue was obtained at least 15 cm from the margin of the carcinoma area. Tissue samples were clamp-frozen in liquid nitrogen and stored at −80 °C until use. The mean age of the patients was 60 years, and there were no differences between males and females. The control group consisted of a cohort of age-matched healthy volunteers. Table 3 lists the clinical characteristics of the study population. The mean age and gender distribution of the cancer patients did not differ from those of the normal control group. Table 4 lists the percentages of tumors corresponding to the different classification grades.

Peripheral mononuclear cells were isolated from 10 mL of heparinized blood by centrifugation over a Lymphoprep (Nycomed) layer at a density of 1.077 g/mL. The cells were then washed twice with RPMI 1640 medium (Gibco BRL) and stored at −80 °C until analysis [71]. The percentage of lymphocytes in the cell suspension was 80%–90%.

Urine and blood samples were obtained from healthy volunteers and from patients at different time points, as indicated in the corresponding figures and tables. All participants received information about the study and provided written informed consent prior to enrolment in the present study, which was performed in accordance with the ethical standards of Spanish law.

### 3.2. Glutathione and Lipid Peroxidation Assay

GSH cell content was determined using the previously described assay [72]. For analysis of oxidized glutathione, samples were treated with N-ethylmaleimide and bathophenanthroline disulfonic acid and were derivatized and analyzed by HPLC, as described previously [73]. MDA was assayed following the method previously described [20]. Protein content was measured using the Bradford method [74].

### 3.3. DNA Isolation and Enzymatic Digestion

DNA from tissues or cells was isolated following the Gupta method [75] with the modification described by Muñiz *et al.*, in, which chloroform isoamyl alcohol (24:1) was used instead of phenol to remove proteins [76]. Isolated DNA was washed twice with 70% ethanol, dried and dissolved in 200 μL of 10 mM Tris-HCl, 0.1 mM EDTA and 100 mM NaCl (pH 7.0) for enzymatic digestion, as described previously [77]. Briefly, 5 μg of DNA/μL (total DNA content 200 μg) was incubated with 100 units of DNase I in 40 μL of Tris/HCl (10 mM) and 10 μL of 0.5 M MgCl_2_ (final concentration: 20 mM) at 37 °C for 1 h. The pH of the reaction mixture was then lowered with 15 μL of 0.5 M sodium acetate (pH 5.1); 10 μL of nuclease P1 (5 units) and 30 μL of 10 mM ZnSO_4_ were added to give a final concentration of 1 mM, and the mixture was incubated for 1 h. After adjusting the pH with 100 μL of 0.4 M Tris/HCl (pH 7.8), followed by addition of 20 μL of alkaline phosphate (3 units), the samples were incubated for 30 min. Enzymes were precipitated with acetone (5 volumes), removed by centrifugation, and the supernatant was evaporated to dryness.

### 3.4. DNA 8-Oxo-7,8-dihydro-2′-deoxyguanosine Separation and Assay

The DNA hydrolysates were dissolved in HPLC grade water and filtered through a 0.2-μm syringe filter before applying the samples to a Waters ODS HPLC column (4.6 mm × 250 mm) 5 μm particle size). A Waters 515 HPLC pump model was used to separate 8-oxo-7,8-dihydro-2′-deoxyguanosine. This separation was carried out using a 5-μm Spherisorb ODS2 column (4.6 mm × 250 mm) with a flow rate of 1 mL/min. The running buffer for 8-oxo-dG from nuclear and mitochondrial DNA was 50 mmol/L potassium phosphate (pH 5.1) in 5% acetonitrile, and the retention time was 7.5 min. Electrochemical detection of the urine samples was performed using an ESA Coulochem II detector equipped with a 5011 high-sensitivity analytical cell (sensitivity of 1 μA), which had coulometric (electrode 1) and amperometric (electrode 2) electrodes linked in series. For the purpose of this assay, the potentials for the two electrodes were set at 0.2 V and 0.4 V, respectively. The amounts of 8-oxo-dG and deoxyguanine (dG) in the DNA digest were measured using electrochemical and UV absorbance detection, respectively, under the elution conditions described previously [78]. Standard samples of dG and 8-oxo-dG were analyzed to ensure their correct separation and to allow identification of those derived from cellular DNA.

### 3.5. Urinary 8-Oxo-7,8-dihydro-2′-deoxyguanosine Isolation and Assay

The first urine in the morning was collected in polyethylene bottles. The volume of the sample was measured, and after agitation, aliquots (2 × 1 mL) of the homogenized urine were kept at −80 °C until further analysis.

The detection of 8-oxo-dG was based on the method described by Brown *et al.* [78]. Briefly, 100 μL of 3 mol/L Tris-EDTA solution (pH 8.6) was added to 1 mL of urine and vortex mixed for 30 s. The solution was then applied to a Bond Elut C18(OH)SPE (3 mL) column that had been pre-equilibrated with 3 mL of methanol and 3 mL of water. The column was washed with 3 mL water, followed by 3 mL of 2.5% acetonitrile and 1.5% methanol in 10 mmol/L borate buffer (pH 7.9). The sample was eluted with 3 mL of the same buffer and applied to a Bond Elut strong cation exchange column (3 mL) that had been pre-equilibrated with 3 mL of methanol and 3 mL of borate buffer (pH 7.9). The 8-oxo-dG was eluted with 2 mL of acetonitrile/methanol in borate buffer and then adjusted to pH 6.0 with 1 mol/L HCl. Then, 4 mL of 50:50 dichloromethane:propane-2-ol was added to 2 mL of eluent and vortex mixed for 30 s. The sample was then centrifuged for 10 min at 3,500 rpm. The upper aqueous layer was caspirated off and 3 mL of organic layer was evaporated to dryness under nitrogen at 50 °C. Finally, the sample was reconstituted in 1 mL of HPLC running buffer without acetonitrile, and 50 μL was injected into the HPLC column.

A Waters 515 HPLC pump model was used to separate 8-oxo-7,8-dihydro-2′-deoxyguanosine. This separation was carried out using a 5-μm Spherisorb ODS2 column (4.6 mm × 250 mm) with a flow rate of 1 mL/min. The buffer used was 50 mmol/L potassium phosphate (pH 5.1) in 5% acetonitrile, and the retention time was 7.5 min. Electrochemical detection of the urine samples was performed using an ESA Coulochem II detector equipped with a 5011 high-sensitivity analytical cell (sensitivity of 1 μA), which had coulometric (electrode 1) and amperometric (electrode 2) electrodes linked in series. The potentials set for the two electrodes were those of nuclear 8-oxo-dG.

To assess the optimization and accuracy of the HPLC-EC assay for the isolation and detection of 8-oxo-dG, appropriate chromatograms of both samples and standards were recorded at the beginning of each working day. The 8-oxo-dG values were expressed as the ratio to creatinine urine concentration given in mmol/mL [79].

### 3.6. mRNA Expression of DNA Repair Enzymes

Total RNA was isolated from frozen tumors, as well as non-tumorous counterparts, using the NucleoSpin RNA/Protein Isolation kit (Macherey-Nagel; Cat. nº 740933.50), which included a DNase incubation step to eliminate contaminating DNA from the isolated RNA, in accordance with the manufacturer’s instructions. *cDNA synthesis:* For reverse transcription reactions (RT), 1 μg of the purified RNA was reverse transcribed using random hexamers with a High-Capacity cDNA Archive kit (Applied Biosystems, P/N: 4322171), according to the manufacturer’s instructions. Reverse transcription conditions consisted of an initial incubation step at 25 °C for 10 min to allow annealing of random hexamers, followed by cDNA synthesis at 37 °C for 120 min and a final inactivation step for 5 min at 95 °C. *Measurement of mRNA levels:* mRNA levels were determined by quantitative real-time PCR analysis using an ABI Prism 7900 HT Fast Real-Time PCR System (Applied Biosystems). Gene-specific primer pairs and probes for hOGG1 (Hs00213454_m1), RAD51 (Hs00153416_m1), MUTYH (Hs01014856_m1), MTH-1 (Hs00159343_m1) and GAPDH (Hs99999905_m1) (Assay-on-demand; Applied Biosystems) were used together with 1× TaqMan^®^ Universal PCR Master Mix (Applied Biosystems, P/N 4304437) and 2 μL of reverse transcribed sample RNA in reaction volumes of 20 μL. Polymerase chain reaction (PCR) conditions were 10 min at 95 °C for enzyme activation, followed by 40 two-step cycles (15 s at 95 °C, 1 min at 60 °C). The levels of GAPDH expression were measured in all samples to normalize gene expression for sample-to-sample differences in RNA input, RNA quality and reverse transcription efficiency. Each sample was analyzed in triplicate, and expression was calculated using the 2^−ΔΔCT^ method [80].

### 3.7. Statistical Analyses

Data were analyzed with SPSS Statistics 19.0 for Windows (SPSS Inc.) and expressed as means ± SD. Mean values of quantitative variables were compared with Student’s *t* test or the Mann–Whitney U test, depending on the size of the group under comparison. The Student’s *t* test was used when comparing patients with control subjects, and the nonparametric Mann–Whitney test was used for comparisons between male and female populations. In all analyses, *p <* 0.05 was taken to indicate statistical significance.

## 4. Conclusions

Gastric carcinoma exhibits high OS levels, where lipid peroxidation and DNA damage are significantly increased compared with normal mucosa. Tumor tissues release significant amounts of 8-oxo-dG, because of its high production and the induction of DNA repair enzymes. The damaged base is concentrated in the urine of affected patients. After gastrectomy, the high levels 8-oxo-dG in urine and PMNC DNA decrease progressively to values close to those found in healthy subjects. This effect is only observed in those patients undergoing efficient surgical resection. Based on our findings, 8-oxo-dG appears to have properties that make it a promising potential tumor marker for use in patients with gastric carcinoma.

## Figures and Tables

**Table 1:** Urinary 8-oxo-7′8-dihydro-2′-deoxyguanosine (8-oxo-dG) and oxidative stress by-products in peripheral mononuclear cells of gastric carcinoma patients *vs.* control subjects.

Metabolites	Controls	Gastric Cancer	*p <* 0.001
8-oxo-dG/10^6^ dG *	4.16 ± 0.73	8.43 ± 1.3	***
8-oxo-dG (nmol/mmol creatinine) **	2.49 ± 1.07	22.29 ± 4.79	***
MDA (nmol/mg protein) *	0.17 ± 0.06	0.52 ± 0.13	***
GSH (nmol/mg protein) *	22.42 ± 3.85	14.00 ± 3.04	***
GSSG (nmol/mg protein) *	4.18 ± 2.02	4.62 ± 1.55	NS
% GSSG/GSH *	18.65%	33.00%	***

Results are expressed as means ± SD of 23 different determinations.

* PMNC values;

** Urinary 8-oxo-dG levels;

*** *p <* 0.001 for the comparison of gastric cancer patients with the control group. NS: not significant.

**Table 2:** Levels of 8-oxo-dG in the urine and peripheral mononuclear cells of patients undergoing surgery without tumor resection.

Metabolites	Basal values	3 Months after Surgery
8-oxo-dG/10^6^ dG *	8.91 ± 1.02	9.37 ± 0.90
8-oxo-dG (nmol/mmol creatinine) **	18.61 ± 2.54	19.98 ± 3.08
MDA (nmol/mg protein) *	0.52 ± 0.09	0.54 ± 0.04
GSH (nmol/mg protein) *	11.54 ± 1.57	15.77 ± 3.97
GSSG (nmol/mg protein) *	4.43 ± 0.51	4.66 ± 0.45
% GSSG/GSH *	38.40%	29.55%

Notes: Results are expressed as means ± SD of three different cases.

* PMNC values;

** Urinary 8-oxo-dG levels.

**Table 3:** Clinical characteristics of the study population.

Clinical Characteristics	Mean Values of Patients and Control Population (m–M)		

	Cancer Patients	Control Subjects	Reference Values
%Female/male	33/67	51/49	-
Mean age	70 (48–90)	60 (36–90)	-
Hb	♀: 10.8 (9.2–12.5)	♀: 13.26 (11.6–16.7)	♀: 11.5–16 g/dL
	♂: 11.44 (8.4–15.2)	♂: 14 (13.6–17)	♂: 13.5–18 g/dL
Hto	♀: 32.3 (27.1–36.9)	♀: 40.1 (35.1–50.6)	♀: 35%–50%
	♂: 34.93 (25.9–56.7)	♂: 42.4 (41.2–51.5)	♂: 40%–54%
Leucocytes	10.33 (3.3–24.1)	5.7 (3.5–11)	3.6–11.5 × 10^9^/L
PCR	5.5 (0.19–19.12)	0.02 (0.01–0.03)	0.00–0.05 mg/dL
CEA	2.61 (0.5–14.4)	-	0.0–3.0 ng/mL
CA19.9	9.28 (0.8–27.5)	-	0.0–35 UI/mL

The results are expressed as means with range in parenthesis. Hb: Hemoglobin; Hto: Hematocrit; CRP: C reactive protein; CEA: Carcinoembryonic antigen; CA19-9: Carbohydrate antigen.

**Table 4:** Percentages of tumor grade distribution in gastric cancer patients.

Tumor Grade	%
I	2.1
I B	8.5
II	27.6
II B	2.1
III A	19.2
III B	6.5
IV	34

Tumors were graded according to the TNM classification.
